# Determinants of COVID-19 Case Fatality Rate in the United States: Spatial Analysis Over One Year of the Pandemic

**DOI:** 10.36469/jheor.2021.22978

**Published:** 2021-05-11

**Authors:** Niranjan J. Kathe, Rajvi J. Wani

**Affiliations:** 1 Senior Manager, Complete HEOR Solutions, North Wales, PA, USA; 2 Real World Evidence Manager, Amgen Canada Inc, Mississauga, ON, Canada

**Keywords:** spatial analysis, health status disparities, social determinants of health, public policy, coronavirus disease 2019, sars-cov-2

## Abstract

**Background:** The United States continues to account for the highest proportion of the global Coronavirus Disease-2019 (COVID-19) cases and deaths. Currently, it is important to contextualize COVID-19 fatality to guide mitigation efforts.

**Objectives:** The objective of this study was to assess the ecological factors (policy, health behaviors, socio-economic, physical environment, and clinical care) associated with COVID-19 case fatality rate (CFR) in the United States.

**Methods:** Data from the New York Times’ COVID-19 repository and the Centers for Disease Control and Prevention Data (01/21/2020 - 02/27/2021) were used. County-level CFR was modeled using the Spatial Durbin model (SDM). The SDM estimates were decomposed into direct and indirect impacts.

**Results:** The study found percent positive for COVID-19 (0.057% point), stringency index (0.014% point), percent diabetic (0.011% point), long-term care beds (log) (0.010% point), premature age-adjusted mortality (log) (0.702 % point), income inequality ratio (0.078% point), social association rate (log) (0.014% point), percent 65 years old and over (0.055% point), and percent African Americans (0.016% point) in a given county were positively associated with its COVID-19 CFR. The study also found food insecurity, long-term beds (log), mental health-care provider (log), workforce in construction, social association rate (log), and percent diabetic of a given county as well as neighboring county were associated with given county’s COVID-19 CFR, indicating significant externalities.

**Conclusion:** The spatial models identified percent positive for COVID-19, stringency index, elderly, college education, race/ethnicity, residential segregation, premature mortality, income inequality, workforce composition, and rurality as important ecological determinants of the geographic disparities in COVID-19 CFR.

## INTRODUCTION

The novel coronavirus of 2019 (COVID-19) pandemic continues to spread in the United States and around the world. The United States, as of April 11, 2021, recorded 31.1 million COVID-19 cases and 561 231 deaths.^1^ Cases and deaths in the United States continue to account for the largest share of global cases (23%) and global deaths (19%).^2^ Containing the COVID-19 pandemic in the United States was challenging due to virus contagion characteristics, its pathophysiology, and socio-political factors.^3^ In response to the pandemic, many states initially adopted safety measures such as mask mandates, social distancing and safety measures for operations of certain businesses.^4,5^ After implementing these restrictive measures including lockdowns, many states rolled back such policies in 2020.^6^ Despite these rollbacks, personal safety measures (mask mandates and social distancing) were continued into the year 2021. However, as of the writing of this study, 14 states in the United States have lifted mask mandates.^7^

Safety measures such as the closure of business establishments, stay-at-home orders, and social distancing mandates severely impacted the economy.^8^ In response to the pandemic, lawmakers passed three stimulus packages and the Coronavirus Aid, Relief, and Economic Security Act (CARES) Act, with additional relief legislation expected.^9,10^ As of April 11, 2021, Moderna, Pfizer, and Johnson & Johnson vaccines have received emergency use authorization by the US Food and Drug Administration and are being rolled out in phases. Other biopharmaceutical companies are conducting clinical trials for 60 COVID-19 vaccine candidates.^11^ The current vaccine rollout will continue to gain momentum while other COVID-19 vaccines may receive market approval in the near future. Despite this progress, experts recommend that the public follow COVID-19 safety measures due to slow initial rollout, uncertainties surrounding COVID-19 vaccines (virus mutations, duration of immunity, real world effectiveness, vaccine uptake, etc.) and vaccine hesitancy.^12,13^ In summary, the US government and the scientific community has undertaken various measures to address the needs of the population during the COVID-19 pandemic. However, the assessment of the impact of ecological contextual factors such as health behaviors, clinical care and burden, socio-economic, and physical environment-related characteristics on the course of the COVID-19 pandemic is necessary. The contextual understanding from such a study is required to gauge whether lock-down measures worked and to what extent. Moreover, the study also aids in identifying high-risk areas for targeting vaccine distribution. Therefore, having a thorough knowledge of these ecological contextual factors is critical to address the public health and economic challenges and prioritize resources.

Studies so far have generated predictive models for growth in COVID-19 incidence and mortality and estimated the impacts of some community-level factors on COVID-19 incidence and mortality. Millett *et al.* and Khanijahani *et al.* focused on assessing the ecological determinants of susceptibility to COVID-19 outcomes among predominantly African American counties, while Fielding-Miller *et al.* and Peters *et al.* performed a similar assessment along the rural-urban continuum.^14–17^ Few studies have assessed the spatial determinants of COVID-19 transmission.^3,18,19^ Recently, Sun *et al.* assessed the relationship between various county-level determinants and COVID-19 incidence while Andersen *et al.* also identified high prevalence clusters.^3,18^ These studies evaluated the incidence and prevalence during the initial phase of the pandemic and limited the analysis to a few county-level and policy-related factors.

Incidence and mortality were critical outcomes in shaping the initial pandemic response to reduce the contagion. However, to reduce fatalities in the current stage of the pandemic, an increasing focus is placed on mitigation strategies such as increasing vaccination rates and continuing safety precautions such as social distancing and mask use. Therefore, COVID-19 case-fatality rate (CFR) is a useful outcomes measure in the current stage of the pandemic. CFR, being less susceptible to testing and reporting biases, also reflects the disease severity. In a study led by author Cao, country-level demographic and socioeconomic characteristics on COVID-19 CFR were presented.^20^ However, to our knowledge, in the United States, no study has estimated the effect of county-level ecological factors, including policy-related factors, on COVID-19 CFR.

The objective of the current study was to assess the impact of county-level ecological factors, using spatial econometric analysis, on the COVID-19 CFR over one year of the pandemic.

## METHODS

### Data source and study design

The current study used county-level COVID-19 confirmed cases and deaths data from the New York Times repository extracted as of February 27, 2021, and included data up until that date.^18^ The study used county-level characteristics from 2020 County Health Rankings data and 2018-2019 Area Health Resource File data. State-level stringency index, percent positive for COVID-19, and social distancing score were obtained from Oxford COVID-19 government response tracker, the COVID Tracking Project, and Unacast, respectively.^4,21–24^ Alaska and Hawaii counties were excluded from the analysis. The US counties ESRI Shapefile was obtained from the US Census Bureau.^25^ The study employed a cross-sectional ecological study design to assess the association between county-level characteristics on the cumulative COVID-19 CFR.

### Outcomes

The county-level US COVID-19 cumulative confirmed cases and deaths data from the New York Times repository was extracted as of February 27, 2021, data up until that date was included.^18^ The COVID-19 cumulative CFR was operationally defined as a ratio of the cumulative COVID-19 deaths by cumulative COVID-19 cases.^20^ The current analysis used CFR as the outcome of interest because it reflects the disease severity, treatment effectiveness, and responsiveness of the health-care system.^20^ Additionally, since the measure is a ratio, compared to incidence rate or mortality rate, the CFR is less sensitive to differences in testing rates across regions.

### Covariates

**Figure 1:**
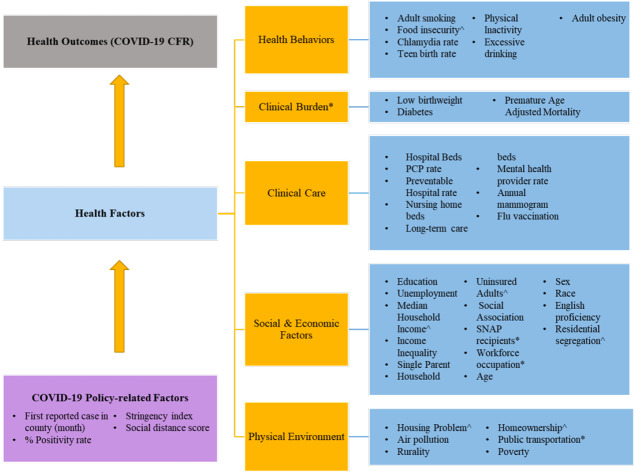
Theoretical Framework Based on the County Health Rankings Model To Establish a Relationship Between COVID-19 Case Fatality Rate and County-level Ecological Factors Abbreviations: CFR, Case fatality rate; SNAP, supplemental nutritional assistance program. ^ indicates that the data was obtained from additional resources and supplementary files of the County Health Rankings^26^; * indicates that the data was obtained from sources other than those in the County Health Rankings data.

The covariates selected within the model to predict the county-level CFR were adapted from the County Health Ranking Framework (CHRF).^26^ The framework categorizes health factors into four sub-categories, namely health behaviors, clinical care, socioeconomic factors, and physical environment (Figure 1). Each of the sub-categories is further divided into individual factors. The CHRF model was used in the current analysis because it provides a well-established theoretical framework for studying ecological determinants of health outcomes. The current analysis augmented the CHRF by including additional covariates based on additional demographic measures from county health ranking, and prior ecological studies on COVID-19. Firstly, an additional sub-category of “clinical burden” was added, which included measures such as low- birth weight, percent diabetes prevalence, and premature age-adjusted mortality. Secondly, nursing home beds, long-term care beds, and total hospital beds were added to the clinical care sub-category.^3^ Thirdly, population characteristics such as rurality, poverty, age distribution, population, population density, supplemental nutritional assistance program (SNAP) eligibility, and percentage of workforce in various occupational categories were added to the social and economic factors sub-category.^3,15,18,27,28^ Additionally, physical environmental factors such as percentage of workers using public transport, were added to the respective sub-category.^28^ Finally, COVID-19 related factors were added to the model, which included month of first infection in the county, positivity rate for COVID-19 at the state-level, social distancing score, and stringency index.^3,4,20–23,29^ The final list of potential county-level covariates and corresponding rationale are described in Table 1. Although the CHRF model assigns weight to each of the components, they were not utilized in the current analysis as no composite rank score was calculated.

**Table 1:** Ecological Factors Used in the Analysis: Source Definitions and Modifications

**Factors**	**Definitions & Rationale**	**Source**
Calendar Month of the First Reported Case in the State	Generated as the month when the first COVID-19 case was reported in the county. Based on the frequency distribution the following categories were created: January, February, March, April, May, and June to December. Month of first reported case has been used in previous studies. Earlier studies have included this variable due to its significant impact on the cumulative cases and deaths during the early phases of the pandemic.^30^	2020-2021 New York Times COVID-19 & Centers for Disease Control and Prevention Data^1^
Percent Positive for COVID-19*	This factor was defined as the proportion of all COVID-19 tests performed that are positive. Positivity rate = (positive tests)/(total tests) x 100%. Percent positivity rate is a proxy measure for extent of under/over testing and has been included to control for impact of geographic differences in testing rates.^31^	The COVID Tracking Project^21^
Social Distance Score	It is defined as the average numerical score based on the following three metrics: Change in average distance traveled compared to a pre-COVID-19 period. Change in visitation to non-essential venues compared to a pre- COVID-19 period. Probability that two devices were in the same place at the same time. During the initial phases of the pandemic, social distances scores were found to be associated with lower COVID-19 mortality.^23^	Unacast^22^
Stringency Index	Composite measure based on 9 response indicators, including school closures, workplace closures, testing policy and travel bans, rescaled to a value from 0 to 100 (100=strictest response). Stringency index has been previously used in ecological research on COVID-19 CFR.^20^	Oxford COVID-19 Government Response Tracker^4^
Percent Adult Smokers	Percentage of adults who are current smokers.	CHRF Data^25^
Percent of Poor or Fair Health	Percentage of adults reporting fair or poor health (age-adjusted).	
Percent Low Birthweight	Percentage of live births with low birthweight (< 2500 grams).	
Teen Birth Rate	Births per 1000 females ages 15-19	
Percent Adult Obesity	Percentage of the adult population (age 20 and older) who report a body mass index greater than or equal to 30 kg/m.^2^	
Percent Excessive Drinking	Percentage of adults reporting binge or heavy drinking.	
Percent Flu Vaccine	Percentage of fee-for-service Medicare enrollees who had an annual flu vaccination.	
Percent with Annual Mammogram	Percentage of female Medicare enrollees having an annual mammogram (age 65- 74).	
Percent Physical Inactivity	Percentage of adults age 20 and over reporting no leisure-time physical activity.	
Income Inequality Ratio	Ratio of household income at the 80th percentile to income at the 20th percentile.	
Primary Care Physicians Rate (log)*	Log of ratio of population to primary care physicians.	
Preventable Hospitalization Rate (log)*	Rate of hospital stays for ambulatory-care sensitive conditions per 100 000 Medicare enrollees.	
Percent Educated at Some College	Percentage of adults aged 25-44 with some post-secondary education.	
Percent Unemployment	Number of people aged 16 years and above unemployed and looking for work.	
Percent Single Parent	Percentage of children who live in single-parent households.	
Social Association Rate	Number of membership associations per 10 000 population.	
Pollution Level: PM 2.5	Average daily density of fine particulate matter in micrograms per cubic meter (PM2.5).	
Percent Severe Housing Problem	Percentage of households with at least 1 of 4 housing problems: overcrowding, high housing costs, lack of kitchen facilities, or lack of plumbing facilities.	
Percent Homeownership	Percentage of occupied housing units that are owned.	
Percent Uninsured Adult	Percentage of adults under age 65 without health insurance.	
Premature Age-adjusted Mortality	Number of deaths among residents under age 75 per 100 000 population (age-adjusted).	
Median Household Income (in Thousands)	The income where half of households in a county earn more and half of households earn less.	
Percent Over 65	Percentage of population aged 65 and older.	
Percent Less Than 18	Percentage of population below 18 years of age.	
Percent Black	Percentage of population who are non-Hispanic African American.	
Percent Native American	Percentage of population who are American Indian or Alaska Native.	
Percent Hispanic	Percentage of population who are Hispanic.	
Percent Female	Percentage of population who are female.	
Segregation Index: White/Non-White	Index of dissimilarity where higher values indicate greater residential segregation between non-White and White county residents.	
Percent Rural	Percentage of population living in a rural area.	
Percent Not Proficient in English	Percentage of population who are not proficient in English.	
Percent Diabetics	Percentage of adults aged 20 and above with diagnosed diabetes.	
Percent with Food Insecurity	Percentage of population who lack adequate access to food.	
Mental Health Provider Rate	Mental health providers per 100 000 population.	
Chlamydia Rate (Log)	Chlamydia cases per 100 000 population	
Percent SNAP Recipients	Percentage of the population on food stamps or SNAP.	2019-2020 AHRF
Percent Public Transportation	Percentage of the population using public transportation.	
Percent of Workforce in Education, Health-care and Social Assistance Field	Percentage of the population in the occupation of education/health-care/social assistance.	
Percent of Workforce in Construction	Percentage of the population who work in construction.	
Percent of Workforce in Manufacturing	Percentage of the population who are manufacturing workers.	
Percent Poverty	Percentage of the population below poverty level.	
Hospital Bed Rates (log)	Number of beds regularly maintained (set up and staffed for use) for inpatients.	
Nursing Home Beds	Number of nursing home unit beds in hospitals.	
Long-term Care Beds (log)	Number of long-term care beds in hospitals.	
Population	Resident population.	
Population Density (log)	Persons per 100 square mile area.	

Abbreviations: AHRF, Area Health Resource Files; CHRF, County Health Ranking Framework; SNAP, supplemental nutritional assistance program.

*These variables are derived or converted for use within the regression model.

### Statistical Analyses

Descriptive univariate statistics of the weighted county-level characteristics were generated. Firstly, all covariates in Table 1, selected as potential covariates, underwent a two-step covariate selection process. In the first step, multicollinearity was assessed and factors with variance inflation factor >7 were excluded.^31^ In the second step, Pearson correlation between remaining factors was tested (**Supplemental Table 1**) and factors with correlation greater than 0.7 were excluded.^20^ All remaining factors were used in regression analysis. Secondly, the presence of spatial correlation was confirmed by performing Moran’s I test for spatial correlation. Two island counties were excluded because spatial regression analysis necessitates that the data contains no island counties. Based on prior research, the LeSage and Pace method was used to determine the best fit spatial regression model.^30^ A first-order queen spatial weight matrix was employed for all spatial models. The queen matrix defines neighbor relationships if the counties either share a border or a vertex. All analysis was performed in RStudio (R) v 4.0.3 (Boston, Massachusetts) and QGIS v 3.16.0 (Berne, Switzerland).

## RESULTS

The final analysis included data from 3101 counties from the mainland United States. Between January 20, 2020 to February 27, 2021, the population-weighted COVID-19 CFR for the mainland United States was 1.82%. Table 2 demonstrates the descriptive statistics of the COVID-19 CFR and county-level determinants, namely, COVID-19 policy-related factors, health behaviors, clinical burden, clinical care, socio-economic, and physical environment factors. Some 2097 counties reported their first case in March 2020. The percent positivity for COVID-19 was 9%. The mean social distance score and stringency index at the county-level was 1.75 and 49.14, respectively. The proportion of adult smokers, those with physical inactivity, obesity, and Medicare enrollees who were administered influenza vaccines were 15%, 23%, 29%, and 46%, respectively. At the county- level, the average ratio of population to primary care physicians was 74 and the average preventable hospitalization rate was 4545 per 10 000 Medicare enrollees. Premature age-adjusted mortality was 342 per 100 000. Among socio-economic factors, the proportion of the workforce in education/health-care/social assistance field, construction, and manufacturing comprised 23%, 7%, and 10% of the population, respectively. Additionally, unemployment was 4%, 12% of adults were uninsured, the mean income inequality ratio was 5 and adults with some college education made up 65% of the population. About 13% were African Americans, 18% Hispanics, 1% Native Americans, 51% females, 33% of the children lived in single-parent households, and 4% of the population was not proficient in English. The percentage of the population older than 65 years and less than 18 years were 16% and 22%, respectively. On average, 19% of counties were rural, the homeownership rate was 64%, 18% used public transportation and 18% of households had severe housing problems. Lastly, population density/100 sq. miles was found to be 2067.

**Table 2:** Descriptive Statistics of Ecological Factors (N=3101)

**Ecological Factors**	**Overall**		**VIF**
Sample size	326 Million (population of 3101 counties)		
	**Mean**	**SD**	
Cases	110 257.75	214 169.92	
Deaths	1991.70	3956.85	
Case fatality rate^#^	1.82	0.82	
**Census Region [n, %] ^**			
Northeast	217.00	7.00	
Midwest	1055.00	33.90	
South	1422.00	45.80	
West	414.00	13.30	
**COVID-19 Policy-related Factors**			
**Calendar Month of the First Reported Case in the State [n, %] ^**	**n**	**%**	2.71
January	6.00	0.20	
February	16.00	0.50	
March	2097.00	67.50	
April	692.00	22.30	
May	133.00	4.30	
June to December	164.00	5.30	
% Positive for COVID-19	9.30	4.10	1.99
Social Distance Score	1.75	0.63	3.30
Stringency Index	49.14	11.21	2.03
**Health Behaviors**			
% Adult Smoking	15.31	3.43	8.85
Food Insecurity	7.83	0.85	3.20
Chlamydia Rate*	520.05	236.40	3.09
% Fair or Poor Health	16.74	3.83	15.65
Teen Birth Rate	22.81	11.11	5.11
% Physical Inactivity	23.31	5.37	2.50
% Excessive Drinking	18.76	2.80	3.09
% Adult Obesity	29.06	5.53	2.06
**Clinical Burden**			
% Low Birthweight	8.07	1.42	3.07
% Diabetic	10.25	2.77	1.91
Premature Age Adjusted Mortality*	341.61	93.01	4.95
**Clinical Care**			
Primary Care Physicians Rate*	75.41	30.36	2.17
Preventable Hospitalization Rate	4545.01	1270.42	1.63
Hospital Beds*	3200.65	5035.30	1.84
Nursing Home Beds*	77.79	187.85	1.13
Long-term Care Beds*	128.89	260.27	1.28
Mental Health Provider Rate*	247.42	161.55	2.09
% With Annual Mammogram	41.15	6.19	2.29
% Influenza Vaccinations	46.11	6.91	2.05
**Socio-economic Factors**			
% SNAP Recipients	12.43	5.92	7.25
% of Workforce in Education/Health-care/Social Assistance Field	23.12	3.92	1.92
% of Workforce in Construction	6.52	1.81	1.53
% of Workforce in Manufacturing	10.23	5.40	2.30
Income Inequality Ratio	4.72	0.78	2.24
% Adults with Some College Education	65.33	9.81	3.97
% Unemployment	3.96	1.12	2.42
% Children in Single-parent Households	33.15	8.68	3.67
Social Association Rate*	9.01	3.60	1.67
% Uninsured Adults	10.25	4.81	3.40
Median Income*	US$65 045.85	US$17 989.94	11.00
% 65 Years Old and Over	16.02	4.04	5.78
% Less Than 18 Years of Age	22.44	2.94	4.70
% African American	12.55	12.56	5.19
% Native Americans	1.24	3.18	2.64
% Hispanic	18.35	17.18	7.80
% Female	50.76	1.23	2.70
% Not Proficient in English	4.41	4.38	4.17
Residential Segregation Non-White/White	37.03	11.27	1.47
% Poverty	13.05	4.82	11.57
**Physical Environment**			
Air Pollution-particulate Matter (2.5)	10.08	2.11	2.94
% Severe Housing Problem	18.09	5.85	2.82
% Rural	18.68	24.06	4.58
% Homeownership	63.77	10.82	3.21
% Public Transportation	18.24	3.95	1.31
Population*	1 172 722.28	1 940 307.84	8.34
Population Density per 100 sq. miles*	2067.26	6497.01	9.12

Abbreviations: SD, Standard Deviation; SNAP, supplemental nutritional assistance program; VIF, Variance Inflation Factor.

# Cumulative case fatality rate calculated as of February 27, 2021.

* VIF was calculated on the log transformed values.

^ Unweighted estimates, as this represents the number of counties

**Figure 2:**
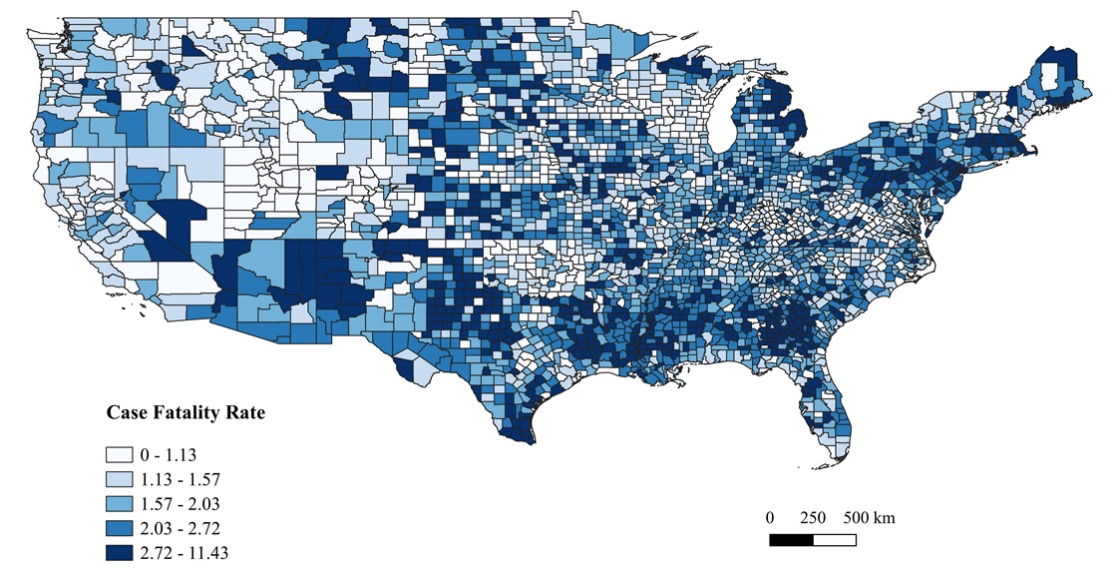
Spatial Distribution of the Cumulative COVID-19 Case Fatality Rate by Quintiles as of February 27, 2021

Figure 2 presents the spatial distribution (quintiles) of COVID-19 CFR. In the West, Washington’s Spokane area and Nevada’s Las Vegas area had high COVID-19 CFR. High COVID-19 CFR clusters were found in border counties of Arizona’s Phoenix area and in New Mexico. In Montana, all major cities such as Helena, Butte, and Billings and along the border of Wyoming (specifically near Yellow Stone National Park) had high COVID-19 CFR. In the Midwestern region, barring the high COVID-19 CFR clusters in Michigan’s Upper Peninsula area, there were many scattered counties with high CFR. The Deep South states of Mississippi, Louisiana, Tennessee, central Alabama, and Arkansas had clusters of high COVID-19 CFR. The Texas panhandle region, the Corpus Christie, Texas, area and the area along the US-Mexico border had clusters of high COVID-19 CFR. In the Northeastern region, high COVID-19 CFR clusters were found between the large parts of Pennsylvania and New Jersey, and in the Boston, Massachusetts, area. Additionally, in Maine, clusters of high COVID-19 CFR were found around Acadia National Park and the northeastern parts of the state.

The first step in the factor selection process identified and excluded percent adult smokers, percent fair/poor health, log population density, log population, percent under poverty, median income, percent eligible for SNAP benefits due to multicollinearity, which is shown in Table 2. Similarly, percent smokers, teen birth rate, percent SNAP eligible, log population, log population density, percent speaking language other than English were excluded based on high correlation with other factors (**Supplemental Table 1**). The presence of spatial autocorrelation was confirmed based on a significant Moran’s I test statistic (Moran’s I=0.256, *P*-value<0.001). The LeSage and Pace method identified that Spatial Durbin Model (SDM) was a better fit to the data compared with other spatial regression models. The significant Rho parameter of the SDM model indicates that (Rho=0.447, *P*-value<0.001), a 1% increase in a neighboring county’s CFR, also results in 0.447% increase in CFR rate in the particular county.

The multivariable SDM results are presented in Table 3. The factors significantly associated with CFR included percent positive for COVID-19 (β=0.059), stringency index (β=0.015), log nursing home beds (β=-0.005), log long-term care beds (β=0.008), percent workforce in construction (β=-0.019), log premature age-adjusted mortality (β=0.711), income inequality ratio (β=0.076), percent population 65 years and older (β=0.055), and percent severe housing problem (β=-0.023). Additionally, the spatial lag term for factors such as percent positive for COVID-19 (lag β=-0.048), stringency index (lag β=- 0.017), social distancing score (lag β=-0.136), adult obesity (lag β=-0.026), log long-term care bed (lag β=0.025), log mental health provider rate (lag β=-0.080), percent workforce in education/health-care/social assistance (lag β=0.018), log social association rate (lag β=0.038), percent female (lag β=-0.057), and percent rurality (lag β=0.005) were also associated with COVID-19 CFR.

**Table 3:** Spatial Durbin Model for the Case Fatality Rates as of February 27, 2021 Using NYT^1^, AHRF^24^, and CHRF^26^ Datasets

**Ecological Factors**	**Spatial Durbin Model**		**Decomposition Estimates**		
	**Estimate**	**Lag Estimate**	**Direct**	**Indirect**	**Total**
**COVID-19 Policy-related Factors**					
Calendar Month of the First Reported Case in the State [n, %]*					
January	*Reference*				
February	-0.253	-0.288	-0.253	-0.673	-0.927
March	-0.375	0.380	-0.345	0.333	-0.011
April	-0.407	0.231	-0.396	0.048	-0.348
May	-0.368	0.026	-0.378	-0.257	-0.635
June to December	-0.586	0.845	-0.521	0.955	0.434
% Positive for COVID-19	0.059 ***	-0.048 ***	0.057 ***	-0.035 **	0.022 **
Social Distance Score	0.062	-0.136 *	0.056	-0.185	-0.129
Stringency Index	0.015 **	-0.017 **	0.014 **	-0.017 **	-0.003
**Health Behaviors**					
Food Insecurity	0.000	0.086	0.011	0.148 *	0.160 *
Chlamydia Rate (log)	-0.034	0.173	-0.024	0.260	0.236
% Physical Inactivity	0.002	-0.009	0.002	-0.013	-0.011
% Excessive Drinking	-0.002	0.025	0.000	0.043	0.043*
% Adult Obesity	-0.009 *	-0.026 *	-0.013 **	-0.054 ***	-0.066 ***
**Clinical Burden**					
% Low Birthweight	-0.022	0.002	-0.023	-0.008	-0.031
% Diabetic	0.008	0.022	0.011 *	0.044 *	0.055 *
Premature Age Adjusted Mortality (log)	0.711 ***	-0.469 *	0.702 ***	-0.234	0.467
**Clinical Care**					
Primary Care Physicians Rate (log)	-0.003	-0.005	-0.003	-0.009	-0.011
Preventable Hospitalization Rate	0.000	0.000	0.000	0.000	0.000
Hospital Beds (log)	0.003	0.001	0.003	0.003	0.006
Nursing Home Beds	-0.005 *	-0.001	-0.005 *	-0.006	-0.011
Long-term Care Beds (log)	0.008 *	0.025 ***	0.010 **	0.048 ***	0.058 ***
Mental Health Provider Rate (log)	-0.001	-0.080 ***	-0.010	-0.136 ***	-0.146 ***
% With Annual Mammogram	-0.001	-0.003	-0.001	-0.004	-0.005
% Influenza Vaccinations^	-0.004	0.003	-0.004	0.002	-0.002
**Socio-economic Factors**					
% of Workforce in Education/Health-care/Social Assistance Field	0.006	0.018 *	0.009	0.036 **	0.045 **
% of Workforce in Construction	-0.019 *	-0.027	-0.024 **	-0.063 *	-0.086 **
% of Workforce in Manufacturing	0.001	0.006	0.002	0.011	0.012
Income Inequality Ratio	0.076 *	-0.063	0.078 **	-0.056	0.022
% Adults with Some College Education	-0.004	-0.001	-0.004 **	-0.005 *	-0.009 **
% Unemployment	0.039	0.040	0.045	0.096	0.141
% Children in Single-parent Households	0.003	0.004	0.003	0.008	0.011
Social Association Rate (log)	0.011	0.038 **	0.014 **	0.073 **	0.087 **
% Uninsured Adults	0.014	0.001	0.015	0.014	0.028 *
% 65 Years Old and Over	0.055 ***	-0.009	0.055 ***	0.024	0.079 **
% Less Than 18 Years of Age	0.016	0.020	0.018	0.044	
% African American	0.006	0.002	0.007 *	0.009	0.016 *
% Native Americans	-0.007	0.000	-0.007	-0.006	-0.013
% Hispanic	-0.003	0.008	-0.002	0.011 *	0.009
% Female	0.018	-0.057 **	0.013	-0.081*	-0.068
Residential Segregation Non-White/White	0.001	0.007 *	0.002	0.013 **	0.015 **
**Physical Environment**					
Air Pollution-particulate Matter (2.5)	0.012	-0.035	0.009	-0.050	-0.041
% Severe Housing Problem	-0.023 ***	0.005	-0.024 ***	-0.009	-0.033
% Rural	-0.001	0.005 *	0.000	0.008 **	0.007 *
% Homeownership	0.004	-0.005	0.004	-0.006	-0.003
% Public Transportation	0.001	0.000	0.001	-0.001	0.000
					
*Rho^*	0.447***				

Abbreviations: AHRF, Area Health Resource Files; NYT, New York Times; CHR: County Health Rankings.

Level of significance: *P-value<0.05, **P-value<0.01, ***P-value<0.001.

^ spatial lag parameter[Bibr ref-57395]

As the β from SDM are not directly interpretable, the estimates from the SDM were decomposed into direct and indirect effects using the Impacts command from *spdep* package as shown in Table 3.^30,32^ Several factors had significant direct impact on the county’s COVID-19 CFR. Firstly, two of the COVID-19 related factors, namely, percent positive for COVID-19 (direct impact: 0.057% point), and stringency index (direct impact: 0.014% point) were positively associated were higher COVID-19 CFR in that county. Secondly, among health behavior related factors, percent adult obesity (direct impact: -0.013% point) was negatively associated with COVID-19 CFR. Thirdly, among the clinical burden and clinical care related factors, percent diabetics (direct impact: 0.011% point), log premature age-adjusted mortality (direct impact: 0.702% point), and log long-term care beds (direct impact: 0.010% point) were positively associated with COVID-19 CFR, while log nursing home beds (direct impact: -0.005% point) was negatively associated with COVID-19 CFR. Several socio-economic factors, importantly, income inequality (direct impact: 0.078% point), log social association rate (direct impact: 0.014% point), and percentage African Americans (direct impact: 0.007% point) were positively associated with COVID-19 CFR, while percentage workforce in construction (direct impact: -0.024% point) and percentage adults with some college education (direct impact: -0.004% point) were negatively associated with COVID-19 CFR.

The decomposition estimates also demonstrated strong indirect effects of spatial lag terms indicating externality associated with ecological factors from surrounding counties on COVID-19 CFR. The directionality of the direct and indirect associations was similar for the majority of the factors. However, some of the factors demonstrated divergent direct and indirect effects on COVID-19 CFR. For illustration, among COVID-19 policy-related factors, both percent positive for COVID-19 (direct: 0.057% point; indirect impact: -0.035% point) and stringency index (direct impact: 0.014% point; indirect impact: -0.017% point) of neighboring counties were negatively associated with COVID-19 CFR in a given county. Interestingly, the magnitude of the indirect associations were larger than the direct associations for the majority of the factors, except for percent 65 years old and over (direct: 0.055% point; indirect: 0.024% point), and percent severe housing problem (direct: -0.024% point; indirect: -0.009% point).

It is noteworthy that for some of the factors, while either the direct or indirect impacts were insignificant, the total impact was found to be significant: for example, food insecurity (total impact: 0.160), log of mental health provider rate (total impact: -0.146% point), percent of workforce in education/health-care/social assistance field (total impact: 0.045% point), percent uninsured adults (total impact: 0.028% point), percent 65 years old and over (total impact: 0.079% point), residential segregation non-White/White (total impact: 0.015% point), and percent rural (total impact: 0.007% point). For percent excessive drinking factor, although the direct and indirect impacts were found to be insignificant, the overall total impact (0.043% point) was significant.

## DISCUSSION

To the best of our knowledge, this is the first spatial analysis study that captured and assessed the COVID-19 CFR through the year 2020-2021 (January 20, 2020 to February 27, 2021) of the COVID-19 pandemic in the United States. The study found that both direct and indirect impacts of food insecurity, diabetes, long-term care beds, and social association rate on COVID-19 CFR was positive. However, only the direct impact of stringency index, premature age-adjusted rate, income inequality ratio, population aged 65 years or more, and African Americans on COVID-19 CFR was significant and positive. Conversely, both the direct and indirect impacts of proportion of adults with obesity, mental health provider rate, workforce in construction, and adults with some college education on COVID-19 CFR was negative. While factors such as nursing home beds and severe housing problem had a negative direct impact on COVID-19 CFR, stringency index and percent females were found to have a negative indirect impact on COVID-19 CFR.

Only one study by Cao *et al* has assessed the ecological determinants of COVID-19 CFR but that study used country-level data.^20^ Similar to our study, Cao *et al* reported that stringency index and diabetes prevalence were associated with higher CFR. Although no US-based studies have assessed an association between county-level ecological factors and COVID-19 CFR, some studies have reported on the ecological determinants on COVID-19 mortality. A few studies have reported high deaths in counties with greater proportions of racial minorities (Hispanics and African Americans) and found results similar to the present study.^15,33^ Stokes *et al* found that greater proportions of income inequality ratio and African American population was associated with high death rates, which is similar to the relationship this study found with CFR as an outcome.^29^

Aside from the similarities with already published literature on COVID-19, this study adds to the literature on ecological determinants of COVID-19 CFR. Firstly, this study demonstrated a positive association between percent positive for COVID-19 and COVID-19 CFR. Percent positive for COVID-19 captures both community level transmission rate and inadequacy of testing.^34^ Hence, increased community-level testing and timely local-level lockdown policies may be needed to improve CFR. Surprisingly, the indirect impact of percent positive for COVID-19 was negative, which warrants further research. Secondly, unlike Cao *et al*, this study found that a higher stringency index in neighboring counties was significantly associated with lower COVID-19 CFR in a given county. However, unlike the indirect association, the direct association between stringency index and COVID-19 CFR was positive due to endogeneity. For illustration, states such as New York and Washington were the early hotspots for COVID-19 cases and deaths.^4^ As a result, due to early lockdown policies that lasted for a long duration, these states had higher stringency index values.^6^ Thirdly, workforce composition in a given county and its surrounding counties were also associated with COVID-19 CFR. Finally, our study found very strong positive association between premature age-adjusted mortality and COVID-19 CFR in a given county. Numerous studies have assessed determinants of premature age-adjusted mortality. Based on the research, public health interventions aimed at reducing premature age-adjusted mortality would also play a vital role in reducing COVID-19 CFR. The study has important limitations. Given the study is cross-sectional and ecological in nature no causal inferences or inferences at the individual level can be made. Although our study included percent positive for COVID-19, there are considerable differences in the testing rates across regions. Relative to incidence and mortality rates, the CFR is less sensitive to testing rates. However, if differential bias in testing of incidence and mortality rates persists then CFR may be biased. Even though stringency index and social distancing scores are included in the study, these measurements were taken at a specific time point. Further, the list of variables is by no means comprehensive and does not include several other factors such as local safety policies (county or city level), and compliance with local and federal prevention guidelines.

## CONCLUSION

The findings of this study are more insightful than the mere coronavirus count meters and data visualizations that depict the spread of the COVID-19 pandemic. The current spatial models incorporated a comprehensive list of factors to ensure that the results, when parsed, offer a multi-faceted explanatory power. For illustration, these models helped identify factors including COVID-19 policy-related factors (stringency index, social distancing score, and percent positive for COVID-19), health behaviors (example: excessive drinking), clinical burden (example: percent diabetic, premature age-adjusted mortality), clinical care (example: mental health provider rates), socio-economic factors (example: race/ethnicity, income inequality, segregation index, education, workforce composition), and physical environment (example: rurality) as some of the important determinants of the geographic disparities in COVID-19 CFR. This study highlights the plausible effect of one’s residential location, vicinity, local state policy and the connectivity to the neighboring counties on COVID-19 CFR. The United States is facing the next set of challenges in limiting fatalities and COVID-19 mutations, while undertaking mass immunization for COVID-19. At this crucial juncture, the current study findings provide guidance on identifying areas at greater risk of COVID-19 CFR.

### CONFLICTS OF INTEREST

The authors declare no competing interests.

### CONSENT FOR PUBLICATION

All authors have consented for this manuscript to be published.

### COMPETING INTERESTS

None.

### AUTHORS’ CONTRIBUTIONS

NK and RW conceived and designed the study; NK and RW collected, cleaned, and curated the data; NK and RW conducted the statistical analysis. NK and RW discussed the results, interpreted the data, and modified the first manuscript. NK and RW supervised the data collection and interpreted the data. Both the authors have read and gave final approval for publication.

## Supplementary Material

Supplementary Material
